# An adaptive optimal ensemble classifier via bagging and rank aggregation with applications to high dimensional data

**DOI:** 10.1186/1471-2105-11-427

**Published:** 2010-08-18

**Authors:** Susmita Datta, Vasyl Pihur, Somnath Datta

**Affiliations:** 1 Department of Bioinformatics and Biostatistics, University of Louisville, Louisville, KY, USA; 2 Department of Biostatistics, Johns Hopkins University, Baltimore, MD, USA

## Abstract

**Background:**

Generally speaking, different classifiers tend to work well for certain types of data and conversely, it is usually not known a priori which algorithm will be optimal in any given classification application. In addition, for most classification problems, selecting the best performing classification algorithm amongst a number of competing algorithms is a difficult task for various reasons. As for example, the order of performance may depend on the performance measure employed for such a comparison. In this work, we present a novel adaptive ensemble classifier constructed by combining bagging and rank aggregation that is capable of adaptively changing its performance depending on the type of data that is being classified. The attractive feature of the proposed classifier is its multi-objective nature where the classification results can be simultaneously optimized with respect to several performance measures, for example, accuracy, sensitivity and specificity. We also show that our somewhat complex strategy has better predictive performance as judged on test samples than a more naive approach that attempts to directly identify the optimal classifier based on the training data performances of the individual classifiers.

**Results:**

We illustrate the proposed method with two simulated and two real-data examples. In all cases, the ensemble classifier performs at the level of the best individual classifier comprising the ensemble or better.

**Conclusions:**

For complex high-dimensional datasets resulting from present day high-throughput experiments, it may be wise to consider a number of classification algorithms combined with dimension reduction techniques rather than a fixed standard algorithm set a priori.

## Background

Sophisticated and advanced supervised learning techniques, such as Neural Networks (NNs) and Support Vector Machines (SVMs), now have to face a legitimate, even though somewhat surprising, competitor in the form of ensemble classifiers. The latter are usually bagging [1], boosting [2], or their variations (arching, wagging) methods which improve the accuracy of "weak" classifiers that individually are no match for NNs and SVMs. Random Forests [3] and Adaboost [4] are the two most notable examples of ensemble tree classifiers that were shown to have superior performance in many circumstances.

Unfortunately, combining "strong" or stable classifiers characterized by small variance, for example, the K-nearest neighbor (KNN) classifiers or SVMs, generally will not result in smaller classification error rates. Thus, there seems to be little or no incentive in running computationally expensive classification methods on random subsets of training data if the final classification accuracy will not improve. Looking from a slightly different angle, it is also naive to expect significant improvements in classifier's accuracy when it is already very close to that of the optimal Bayes classifier which cannot be improved upon. However, in a real-world problem, neither the *optimal *classification accuracy nor the *true *accuracy of any individual classifier are known and it is rather difficult to determine which classification algorithm does have the best accuracy rates when applied to specific observed training data.

In a recent classification competition that took place in the Netherlands several research groups were invited to build predictive models for breast cancer diagnosis based on proteomic mass spectrometry data [5]. Their models were objectively compared on separate testing data which were kept private before the competition. Interestingly enough, despite the "controlled" environment and the objectivity in assessing the results, no single group emerged as the winner. This was in part due to the difficulty in determining the "best" model which highly depended on what performance measure was used (accuracy, sensitivity or specificity). The over-all conclusion made after the fact was that no single classification algorithm was the best and that algorithms' performance highly correlated with user's sophistication and interaction with the method (setting tuning parameters, feature selection and so on). In general, since no single classification algorithm performs optimally for all types of data, it is desirable to create an ensemble classifier consisting of commonly used "good" individual classification algorithms which would adaptively change its performance depending on the type of data to that of the best performing individual classifier.

In this work, we propose a novel adaptive ensemble classification method which internally makes use of several existing classification algorithms (user selectable) and combines them in a flexible way to adaptively produce results, at least, as good as the best classification algorithm from among those that comprise the ensemble. The proposed method is inspired by a combination of bagging and rank aggregation. In our earlier work, the latter was successfully applied in the context of aggregating clustering validation measures [6]. Out-of-bag (OOB) samples play a crucial role in the estimation of classification performance rates which are then aggregated over through rank aggregation to obtain the locally best performing classifier $A_{\left(1\right)}^{j}$ given the *j*^*th *^bootstrap sample.

Being an *ensemble *classification algorithm, the proposed classifier differs from traditional ensemble classifiers in at least two aspects. The first notable feature is its *adaptive *nature, which introduces enough flexibility for the classifier to exhibit consistently good performance on many different types of data. The second aspect is the *multi-objective *approach to classification where the resulting classification model is optimized with respect to several performance measures simultaneously through weighted rank aggregation. The proposed adaptive multi-objective ensemble classifier brings together several highly desirable properties at the expense of increased computational times.

The manuscript is organized as follows. The Results section presents two simulated (threenorm and simulated microarray data) and two real-data examples (breast cancer microarray data and prostate cancer proteomics mass spectrometry data) which clearly demonstrate the utility of the proposed method. This is followed by a discussion and general comments. In the Methods section, we describe the construction of the adaptive ensemble classifier and introduce some common classification algorithms and dimension reduction techniques that we use for demonstrating the ensemble classifier.

## Results and Discussion

### Performance on Simulated Data

#### Threenorm data

This is a *d*-dimensional data with two class labels. The first class is generated with equal probability from either one of the two normal distributions *MN*({*a*, *a*, ..., *a*}, *I*) and *MN*({-*a*,-*a*, ..., -*a*}, *I*) (*I *denotes the identity matrix), and the second class is generated from a multivariate normal distribution *MN*({*a*,-*a*, *a*,-*a*, ... *a*,-*a*}, *I*). $a=\frac{2}{\sqrt{d}}$ depends on the number of features *d*. This benchmark dataset was introduced in [7] and is available in the *mlbench *R package.

We generate 100 training samples from 1000- dimensional threenorm distribution. Eight individual classification algorithms, including Support Vector Machines (SVM), Lasso Penalized Logistic Regression (PLR), Random Forest (RF), Partial Least Squares followed by RF (PLS + RF), Linear Discriminant Analysis (PLS + LDA) and Quadratic Discriminant Analysis (PLS + QDA), Principal Component Analysis followed by Linear Discriminant Analysis (PCA + LDA), and PLS, as well as our proposed ensemble classifier are trained on these data and their performance is assessed using a different testing set consisting of a different set of 100 samples. We also included a more direct but perhaps somewhat naive ensemble, called the greedy ensemble. Internally, the performance is optimized with respect to three performance measures, namely, accuracy, sensitivity and specificity. This procedure is repeated 100 times and average accuracy, sensitivity, specificity and area under the curve (AUC) along with corresponding standard errors are reported in Table 1. Both the PCA and PLS are used with five components (arbitrarily selected) and the number of bootstrap samples was set to 101. For the RF and SVM, default parameters in corresponding R implementations were used. Selection of meta-parameters could be also based on a prior cross-validation of an individual classifier as well as the inclusion of several different choices as separate "algorithms" within the ensemble itself, in a way analogous to including different kernels for the SVM for the simulated microarray data below.

**Table 1:** Threenorm simulation data

	Accuracy	Sensitivity	Specificity	AUC
SVM	0.451900	0.468200	0.435600	0.429016
	(0.00988)	(0.02144)	(0.02314)	(0.01318)
RF	0.562200	0.557600	0.566800	0.591170
	(0.00540)	(0.00853)	(0.00806)	(0.00635)
PLS + LDA	0.610000	0.608000	0.612000	0.610032
	(0.00561)	(0.00860)	(0.00797)	(0.00561)
PCA + LDA	0.503600	0.501800	0.505400	0.505236
	(0.00617)	(0.00674)	(0.00680)	(0.00753)
PLS + RF	0.612200	0.586400	0.638000	0.648102
	(0.00506)	(0.01250)	(0.01198)	(0.00595)
PLS + QDA	0.607500	0.617200	0.597800	0.607500
	(0.00577)	(0.01142)	(0.01218)	(0.00577)
PLR	0.540800	0.538000	0.543600	0.557342
	(0.00459)	(0.00819)	(0.00804)	(0.00553)
PLS	0.600300	0.600400	0.600200	0.647896
	(0.00542)	(0.01319)	(0.01361)	(0.00609)
Greedy	0.596600	0.581800	0.611400	0.621590
	(0.00559)	(0.01117)	(0.01045)	(0.00657)
Ensemble	0.613000	0.606200	0.619800	0.653700
	(0.00563)	(0.00823)	(0.00729)	(0.00587)

Average accuracy, sensitivity, specificity and AUC for 100 datasets from the threenorm data with *N *= 100 and *d *= 1000. Standard errors are reported in parentheses.

For these datasets, the algorithm which uses the PCA for dimension reduction, PCA + LDA, and SVM clearly underperform in comparison to the other six individual classifiers. It is interesting to note that PLS-based classification methods exhibit very strong performances comparable to that of RF. Overall, PLS + RF has the best scores among the eight individual classifiers for the three out of four performance measures, while PLS + QDA has the best sensitivity rate. The ensemble classifier's accuracy, sensitivity and specificity are very similar to those of the top performing individual classifiers. The greedy ensemble performs well but its overall performance is consistently inferior to the proposed ensemble classifier, albeit by not much. Standard errors for the ensemble classifier are also a little smaller than the standard errors for the greedy algorithm. The AUC scores were not used in the aggregation process where we optimized with respect to accuracy, sensitivity and specificity. So these scores are valid indicators of the performance which take into consideration both sensitivity and specificity. The ensemble classifier has the largest AUC score.

#### Simulated microarray data

The simulation scheme incorporates the simplest model for microarray data where *d *= 5000 individual probes are generated independently of each other from *N*(*μ*, 1). 90% of probes do not differ between cases and controls and their expression values come from normal distribution with unit variance. The other 10% of probes have different means between cases whose expression values are generated from *N*(.3, 1) and controls which are generated from *N*(0, 1).

50 training and testing datasets were generated and average accuracy, sensitivity, specificity and AUC were computed for the testing data which are shown in Table 2.

**Table 2:** Simulated microarray data

	Accuracy	Sensitivity	Specificity	AUC
linear SVM	0.902200	0.907600	0.896800	0.967464
	(0.00451)	(0.00683)	(0.00679)	(0.00216)
polynomial SVM	0.506200	0.716400	0.296000	0.498772
	(0.00383)	(0.05493)	(0.05477)	(0.00640)
radial SVM	0.773200	0.882000	0.664400	0.833576
	(0.03090)	(0.02851)	(0.04473)	(0.03750)
sigmoid SVM	0.905000	0.910400	0.899600	0.968472
	(0.00432)	(0.00655)	(0.00581)	(0.00210)
greedy	0.671400	0.807200	0.535600	0.702040
	(0.04177)	(0.03811)	(0.05508)	(0.05016)
Ensemble	0.900600	0.902400	0.898800	0.968156
	(0.00366)	(0.00661)	(0.00592)	(0.00213)

Average accuracy, sensitivity, specificity and AUC for 50 datasets from the simulated microarray data with *N *= 100 and *d *= 5000. Standard errors are reported in parentheses. A single SVM classifier was used with four different kernel settings.

To illustrate the point that the proposed ensemble algorithm can be used with any combination of individual classifiers or even same classifiers with different settings of tuning parameters, for this example, we selected the SVM algorithm with four different kernel parameters: linear, polynomial, radial and sigmoid. The default settings for each of the kernels were used. The ensemble classifier performs similarly to the SVM with the sigmoid kernel and clearly outperforms the greedy algorithm.

### Performance on real data

#### Breast cancer microarray data

These data are publicly available through the GEO database with the accession number GSE16443 [8] and were collected with the purpose of determining the potential of gene expression profiling of peripheral blood cells for early detection of blood cancer. It consists of 130 samples with 67 cases and 63 controls.

For our classification purposes we downloaded the normalized data which contains 11217 probes. Six individual classification algorithms were selected and they are listed in Table 3. To estimate performance scores, we performed double cross-validation where the inner cross-validation was used to select the best performing classification algorithm based on aggregated validation measures (accuracy, sensitivity and specificity) followed by the outer 10-fold cross-validation. The results are reported in Table 3. In contrast to our simulated data, we need to resort to 10-fold cross-validation to estimate the performance measures in real dataset such as these. Unlike earlier scenarios, none of the individual algorithms appears to outperform all others according to all performance measures which include accuracy, sensitivity and specificity. PLS + QDA has the best estimated accuracy of .64 and the best estimated sensitivity rate of .70, while PLS + LDA has the best estimated specificity rate of .69. While the ensemble classifier falls a little short on all these counts, it is clearly optimized with respect to all three measures and the largest AUC when compared to all individual estimates of AUC demonstrates that.

**Table 3:** Breast cancer microarray data

	Accuracy	Sensitivity	Specificity	AUC	Count
SVM	0.5846	0.6679	0.5525	0.6845	168
PLR	0.6154	0.6859	0.5706	0.6503	197
PLS + RF	0.6077	0.6615	0.5562	0.6498	170
PLS + LDA	0.6846	0.6744	0.6887	0.6826	305
PLS + QDA	0.6462	0.7063	0.5799	0.6871	78
PCA + QDA	0.4692	0.3127	0.6645	0.5401	92
Ensemble	0.6385	0.6563	0.6227	0.7108	

Average of 10-fold cross validation for the breast cancer microarray data. The number of bootstraps *N *= 101. The count column shows the number of times a particular individual algorithm was a locally "best" performing classifier across all 10 folds.

#### Proteomics data

To assess the predictive power of proteomic patterns in screening for all stages of ovarian cancer, [9] carried out a case-control SELDI (surface-enhanced laser desorption and ionization time-of-flight) study with 100 cases and 100 controls. Each spectrum was composed of 15200 intensities corresponding to m/z values on a range from 0 to 20000. Subsequently, the scientific findings of this paper were questioned by other researchers [10,11] who argued that the discriminatory signals in this dataset may not be biological in nature. However, our use of this dataset for the purpose of an illustrative example of the comparative classification ability of our ensemble classifier is still valid.

For this illustration, we applied five classification algorithms to these high-dimensional data and our proposed ensemble classifier with the number of bootstrap samples equal to 101. Once again, the internal optimization of the ensemble classifier was performed with respect to accuracy, sensitivity and specificity.

 Similar to the microarray data, we implemented an external 5-fold cross-validation and the average performance scores are reported in Table 4. In this example, PLS + LDA has the largest overall accuracy and sensitivity, while SVM has the largest specificity and RF takes the top spot according to AUC. Please note that our ensemble method does have performance scores very close to those of top classifiers in each performance category. 

**Table 4:** Proteomics ovarian cancer data

	Accuracy	Sensitivity	Specificity	AUC
RF	0.9550	0.9639	0.9520	0.9924
SVM	0.9350	0.9021	0.9731	0.9795
PLS + RF	0.9050	0.9040	0.9029	0.9703
PLS + LDA	0.9600	0.9639	0.9624	0.9784
PLS + QDA	0.9550	0.9539	0.9648	0.9781
Ensemble	0.9650	0.9639	0.9711	0.9871

Averages of 5-fold cross validation for the proteomics ovarian cancer data.

## Conclusions and Discussion

For complex high dimensional datasets resulting from present day high throughput experiments, it may be wise to consider a number of reputable classification algorithms combined with dimension reduction techniques rather than a single standard algorithm. The proposed classification strategy borrows elements from bagging and rank aggregation to create an ensemble classifier optimized with respect to several objective performance functions. The ensemble classifier is capable of adaptively adjusting its performance depending on the data, reaching the performance levels of the best performing individual classifier without explicitly knowing which one it is.

For a number of different data that we considered here, the best performing method according to any particular performance measure changes from one dataset to another. In some cases, if the three performance measures are considered (accuracy, sensitivity and specificity), it is not even clear what the best algorithm is. In such cases, the ensemble method appears to be optimized with respect to all three measures which can be concluded from it having the largest (or very close to the largest) AUC scores.

The biggest drawback of the proposed ensemble classifier is the computational time it takes to fit *M *classification algorithms on *N *bootstrap samples. In addition, rank aggregation may also take considerable time if *M *is large. We have implemented the procedure in R using available classification routines to build the ensemble classifier. On a workstation with an AMD Athlon 64 X2 4000+ Dual Core processor and 4GB of memory, it takes about five hours to run the ensemble classifier with 10-fold cross-validation on the breast cancer microarray data. For a slightly larger proteomics example, 101 boot-strap samples with 5-fold external cross-validation take approximately 17 hours to complete which is mainly due to the size of the dataset (15200 covariates) where even individual classifiers take considerable time to build their models (in particular RF). Computing variable importance is also very computationally intensive but is not essential for building an ensemble classifier. It should be noted that it is relatively easy to parallelize the ensemble classifier which would reduce the computing times dramatically if run on a grid or cluster. If a cluster is not available and one is dealing with high-dimensional data, feature selection is commonly performed prior to running the ensemble classifier to reduce the dimensionality of the data to more manageable sizes. As with any classification algorithm, feature selection should be done with great caution. If any cross-validation procedure is implemented, feature selection should be performed separately for every training set to avoid over-optimistic accuracy estimates [12]. In simulation examples, the greedy algorithm performs somewhat worse than the proposed ensemble classifier which is why it was not considered further for real data illustrations. Not surprisingly, it still demonstrates good performance overall. Generally speaking, it also takes less time to execute because it is based on a *k*-fold cross-validation where *k *is relatively small (usually between 5 and 10) instead of a computationally intensive bootstrap sampling where *N *is usually much larger. Also, the greedy algorithm performs a single rank aggregation, while the proposed ensemble classifier performs *N *of them, one for each bootstrap sample. For a small number of individual classification algorithms, *M *≤ 10 or so, this does not add a substantial computational burden on the ensemble classifier. If one is willing to sacrifice on the number of bootstrap samples *N*, then the running times of the two algorithms not too different.

For the illustration purposes, we used some common classification algorithms and dimension reduction techniques in this paper. Obviously, many other individual classifiers and dimension reduction techniques could be incorporated into the ensemble. For example, one could select features based on the importance scores returned by the Random Forests to reduce the dimension of the data [13,14] and follow that with any classification algorithm. Also, performance measures are not limited to the commonly used accuracy, sensitivity and specificity. If moving beyond a binary classification problem, sensitivity and specificity can easily be replaced by class-specific accuracies. Still other performance measures are available which are functions of class assignment probabilities, for example the Brier score [15] and the kappa statistic [16]. It is beyond the scope of this paper to discuss or make specific recommendation as to which component classification algorithms are to be included in the ensemble and the selection and setting of tuning parameters for individual classifiers. We have a few more illustrations of the our ensemble classifier on the supplementary web-site at http://www.somnathdatta.org/Supp/ensemble/.

Following the standard bagging principle we have used simple random sampling for generating our bootstrap samples. Note that a certain bootstrap sample may not include all the classes and thus prediction using these samples will also be limited to these classes. As pointed out by one of the reviewers, this may appear to be problematic, especially, in situations when one or more of the classes are rare in the overall population. Since a large number of bootstrap samples is taken, the principle of unbiasedness still applies to the overall aggregation; nevertheless, this may lead to inefficiencies. Alternative sampling strategies (e.g., sampling separately from each class to match the original training data, non-uniform probability sampling related to the class prevalence, etc) that are more efficient can be considered in such situations. Subsequent aggregation should then be done through appropriate reweighing of the individual predictions. A detailed investigation of such alternative resampling strategies is beyond the scope of this paper and will be explored elsewhere.

## Methods

### Construction of an adaptive ensemble classifier

The goal of any classification problem is to train classifiers on the training data, *X*_(*n × p*)_, with known class labels *y *={*y*_1 _,..., *y*_*n*_} to be able to accurately predict class labels $\overset{\wedge }{y}=\{{\overset{\wedge }{y}}_{1},...,{\overset{\wedge }{y}}_{r}\}$ from the new testing data $X_{\left(r\times p\right)}^{*}$. Here, both *n *and *r *are the number of samples in training and testing data respectively, and *p *is the number of predictors (features). Suppose one considers *M *classification algorithms, *A*_1 _,..., *A*_*M*_, with the true, but unknown, classification error rates of *e*_1 _, ..., *e*_*M*_. By drawing random bootstrap samples [17] from the training data {*X*_(*n *× *p*)_, *y*_(*n *× 1)_} and training each classifier on them, it is possible to build a number of "independent" models which can then be combined or averaged in some meaningful fashion. Majority voting is a common solution to model averaging but more complex schemes have been proposed in the literature [18-20].

To build an ensemble classifier, we combine bootstrap aggregation (bagging) and rank aggregation in a single procedure. Bagging is one of the first model averaging approaches to classification. The idea behind bagging is that averaging models will reduce variance and improve the accuracy of "weak" classifiers. "Weak" classifiers are defined as classifiers whose final predictions change drastically with little changes to training data. In bagging, we repeatedly sample from a training set using simple random sampling with replacement. For each bootstrap sample, a single "weak" classifier is trained. These classifiers are then used to predict class labels on testing data and the class that obtains the majority of the votes wins.

We adopt the same strategy for building our adaptive ensemble classifier with the exception that we will train several (*M*) classifiers on each bootstrap sample. A classifier with the best performance on OOB samples will be kept and used for prediction on testing data. The second major difference lies in the fact that we do not seek to improve upon accuracies of individual classifiers. "Strong" classifiers that we are using are quite difficult to improve and the goal here is to create an ensemble classifier whose performance is very close to that of the best performing individual classifier which is not known *apriori*. Our procedure is adaptive in a sense that it will dynamically adjust its performance to reflect the performance of the *best *individual method used for any given classification problem.

How well a classification method can predict class labels is quantified by common performance measures such as an overall accuracy, and sensitivity/specificity for binary classification problems (Table 5). A Receiver Operating Characteristic (ROC) curve is a graphical tool for assessing the performance of a binary classifier. It is a plot of sensitivity versus 1-specificity computed for varying thresholds of class probabilities. The area under the curve (AUC) puts a numerical score which is equal to 1 for a perfect classification at all threshold levels and is around .5 for a random guess classification. Classifiers with AUC smaller than .5 are considered inferior to random guessing [21].

**Table 5:** Confusion matrix

	True			
		Class 1	Class 0	Total
Predicted	Class 1	a	b	a + b
	Class 0	c	d	c + d
	
	Total	a + c	b + d	a + b + c + d

Confusion matrix from which many performance measures (accuracy, sensitivity, specificity) can be computed.

In many classification settings, in medical applications domain in particular, the overall prediction accuracy may not be the most important performance assessment measure. Depending on a condition or treatment, making one type of a misclassification can be much more undesirable than the other. For binary prediction problems, sometimes large sensitivity and/or specificity rates are highly sought after in addition to the overall accuracy. Thus, it is important under many circumstances to consider several performance measures simultaneously. Explicit multi-objective optimization is very attractive and the construction of a classifier which would have an optimal performance according to all performance measures, perhaps weighted according to the degree of their importance, is very desirable.

It is straightforward to determine which classification algorithm performs the best if a single performance measure is considered. For example, if overall accuracy is the only measure under consideration, a classifier with the largest accuracy on OOB samples will be kept. However, if several measures are of interest, determining which classifier to keep becomes a challenging problem in itself, since now we are interested in a classifier whose performance is optimized with respect to all performance measures.

Assume we want our classification model to have high sensitivity rate in addition to high overall accuracy rate. In the proposed ensemble classifier, this multi-objective optimization is carried out via the weighted rank aggregation. Each performance measure ranks classification algorithms according to their performance under that particular measure. The ordered lists of classification algorithms, *L_1_*, ..., *L_K_*, where *K *is the number of performance measures under consideration, are then aggregated to produce a single combined list which ranks algorithms according to their performance under all *K *measures simultaneously. The objective function is defined as

(1) $$\Phi (\delta )=\sum_{i=1}^{K}w_{i}d(\delta ,L_{i}),$$

where *δ *is any valid ordered list of classification algorithms of size *M*, *d *is a distance function that measures the "closeness" between any two ordered lists and *w*_*i *_is a weight factor associated with each performance measure. The two most common distance functions used in the literature are Spearman footrule distance and Kendall's tau distance [22].

Here, we perform the rank aggregation in which the minimization of Φ can be carried out using a brute force approach if *M *is relatively small (< 8). For larger optimization problems, many combinatorial optimization algorithms could be adapted. We use the Cross-Entropy [23] and/or Genetic [24] algorithms which are described in the context of rank aggregation in [25]. The weights *w*_*i *_play an important role in aggregation allowing for greater flexibility. If highly sensitive classification is needed, more weight can be put on sensitivity and algorithms having higher sensitivity will be ranked higher by the aggregation scheme.

Next we present a step-by-step procedure for building an adaptive ensemble classifier. Assume we are given training data consisting of *n *samples {*X*_(*n *× *p*_), *y*_(*n *× 1)_}.

1. **Initialization**. Set *N*, the number of bootstrap samples to draw. Let *j *= 1. Select the *M *classification algorithms along with *K *performance measures to be optimized.

2. **Sampling**. Draw the *j*^*th *^bootstrap sample of size *n *from training samples using simple random sampling with replacement to obtain $\left\{X_{j}^{*},y_{j}^{*}\right\}$. Sampling is repeated until samples from all classes are present in a training set. Please note that some samples will be repeated more than once, while others will be left out of the bootstrap sample. Samples which are left out of the bootstrap samples are called out-of-bag (OOB) samples.

3. **Classification**. Using the *j*^*th *^bootstrap sample train the *M *classifiers.

4. **Performance assessment**. The *M *models fitted in the Classification step are then used to predict class labels on the OOB cases which were not included into the *j*^*th *^bootstrap sample, $\left\{X_{j}^{oob^{*}},y_{j}^{oob^{*}}\right\}$. Since the true class labels are known, we can compute the *K *performance measures. Each performance measure will rank classification algorithms according to their performance under that measure, producing *K *ordered lists of size *M*, *L*_1 _,..., *L*_*K*_.

5. **Rank aggregation**. The ordered lists *L*_1 _,..., *L*_*K *_are aggregated using the weighted rank aggregation procedure which determines the best performing classification algorithm $A_{\left(1\right)}^{j}$. Steps Sampling through Rank aggregation are repeated *N *times.

**Figure 1:**
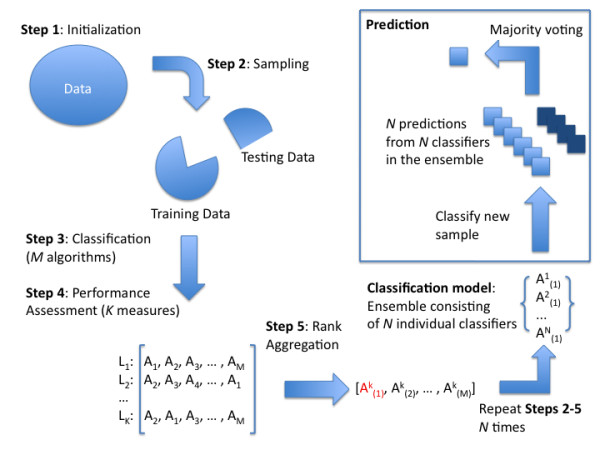
Workflow of our ensemble classifier.

The flowchart depicting both building the ensemble classifier as well as using it to predict new samples is shown in Figure 1.

In essence, bagging takes the form of a nested cross-validation in our procedure which is used to select the best performing algorithm for each bootstrap sample. The outer cross-validation can be added to estimate performance rates and we use a *k*-fold cross-validation scheme for that purpose (see the breast cancer microarray data results).

To predict new cases, the ensemble algorithm runs them through the *N *fitted models. These will likely be of different types, unlike classification trees in bagging, since different classification algorithms will exhibit the "best" local performance. Each model casts a "vote" as to which class a particular sample belongs to. The final prediction is based on the majority vote and the class label with the most votes wins. A more detailed description of the prediction algorithm is given below.

•**Individual Predictions**. Use the *N *"best" individual models, $A_{\left(1\right)}^{1},...,\;A_{\left(1\right)}^{N}$, built on training data for each bootstrap sample to make *N *class prediction for each sample. Given a new sample *x*_*(p × 1)*_, let ${\overset{\wedge }{y}}_{1},\;...,\;{\overset{\wedge }{y}}_{N}$ denote *N *class predictions from *N *individual classifiers.

•**Majority voting**. The final classification is based on the most frequent class among the *N *predicted class labels, also known as majority voting defined as

$$\arg\underset{c}{\max}\sum_{i=1}^{N}I({\overset{\wedge }{y}}_{i}=c),$$

where *N *is the number of bootstrap samples and *c *is one of the class labels.

•**Class probabilities**. Compute the probability of belonging to a particular class *c *by a simple proportion of votes for that class

$$P(C=c|X=x)=\frac{1}{N}\sum_{i=1}^{N}I({\overset{\wedge }{y}}_{i}=c).$$

#### Variable importance

Some classical classification algorithms allow for a formal statistical inference about the contribution of each predictor to the classification. For high-dimensional data, variable importance becomes a challenge as most classical methodologies fail to cope with high dimensionality. Computationally intensive nonparametric methods based on permutations can come to rescue in those situations. In Random Forests, Breiman introduced a permutation-based variable importance measure which we adapt for our ensemble classifier [3].

In the context of Random Forests where many classification trees are grown, the performance is assessed by classifying the OOB samples *X*^*oob*^. To assess the importance of the *m*^*th *^variable, Breiman proposes to randomly permute the *m*^*th *^variable values in the OOB samples, $X_{\left(m\right)}^{oob}$, and then classify the OOB samples with one permuted variable using the built trees. Intuitively, if misclassification error rate increases rather dramatically when compared to the non-permuted samples, the variable is quite important. The formal measure that captures the raw importance of a variable *m *is defined as the average difference between the error rates when using non-permuted and permuted OOB data on all *N *trees

$$I_{m}=\frac{1}{N}\sum_{i=1}^{N}\left(e_{i}^{X_{\left(m\right)}^{oob}}-e_{i}^{X^{oob}}\right).$$

This idea can be easily adapted to the ensemble classifier with the exception that instead of averaging across the *N *trees, we average the misclassification error across locally best performing algorithms as selected through the rank aggregation.

#### An alternative greedy ensemble approach

In addition to the proposed ensemble classifier, we also consider an alternative greedy ensemble classification algorithm (greedy), which is more naive and direct. Here, we simply determine the best performing individual classifier using *k*-fold cross-validation where performance scores for each performance measure and each individual algorithms are first averaged across the *k *folds and then aggregated over the performance measures using the weighted rank aggregation. The top performing individual classifier is used to predict testing cases, so no model averaging is necessary.

1. **Data Management**. Split training data into *k *folds.

2. **Classification**. Using the *i*^*th *^fold (*i *= 1, ..., *k*) for testing, train *M *classifiers on the remaining *k *- 1 folds and compute *K *performance measures for each individual classification algorithm.

3. **Averaging**. Average the performance scores across the *k *folds.

4. **Rank Aggregation**. Using the weighted rank aggregation procedure, determine the "best" performing classification algorithm.

We implement the greedy ensemble to compare its performance to the proposed adaptive ensemble classifier. We expect the greedy ensemble to possibly overfit the training data and, therefore, have an inferior performance with the test data.

### Some common classification algorithms used in our ensembles

Classification algorithms in both statistical and machine learning literatures provide researchers with a very broad set of tools for discriminatory analysis [26]. They range from fairly simple ones, such as the K-nearest neighbor classifier to the advanced and sophisticated Support Vector Machines. Which classification algorithm should be used in any specific case highly depends on the nature of data under consideration and its performance is usually sensitive to the selection of its tuning parameter. In the next several sections we will briefly describe several most common classification algorithms which are particularly popular in bioinformatics. These algorithms in combination with dimension reduction techniques will be used as component classifiers for our ensemble classifier. Of course, in principle, the user could use any set of classifiers in constructing the ensemble.

#### Logistic regression and penalized logistic regression

Logistic regression (LR) is perhaps the most widely used model when dealing with binary outcomes [27]. In the context of classification it applies to a two-class situation. It models the probability of a success (here denoted as *C *= 1) using the following relationship

$$P(C=1|X=x)=\frac{exp(\beta_{0}+\beta^{\prime }x)}{1+exp(\beta_{0}+\beta^{\prime }x)},$$

where *β*_0 _and *β *are the parameters maximizing the log-likelihood function. The model is usually equivalently expressed as a relationship between a linear function of data and the logit transformation of the probability of a success

$$\log\left(\frac{P(C=1|X=x)}{1-P(C=1|X=x)}\right)=\beta_{0}+\beta^{\prime }x.$$

Parameters in this model are estimated via the Newton-Raphson algorithm, an iterative numerical technique used for solving nonlinear systems of equations. As with most classical statistical techniques, the maximum number of parameters that can be reliably estimated should be small when compared to the number of samples in the data. When the number of features is larger than the number of samples as is usually the case for genomic and proteomic data, feature selection has to be performed to reduce the dimensionality of the data. An alternative approach is to use a penalized logistic regression (PLR) where a penalty is imposed on the log-likelihood function corresponding to the logistic regression

$$\ell^{*}(\beta )=\ell (\beta )-\lambda J(\beta ).$$

Here, λ is the tuning parameter controlling how much penalty should be applied, and *J*(*β*) is the penalty term which usually takes the two common forms: ridge penalty defined as ${\sum_{i=1}^{p}\beta }_{i}^{2}$ and the lasso penalty defined as $\sum_{i=1}^{p}|\beta_{i}|$. Due to the penalty term, many of the estimated parameters will be close to 0.

#### Linear and Quadratic Discriminant Analysis

Linear Discriminant Analysis (LDA) is one of the classical statistical classification techniques originally proposed by [28]. The LDA can be derived via a probability model by assuming that each class *c *has a multivariate normal distribution with mean *μ*_*c *_and a common covariance matrix ∑. Let π_*c *_be the prior probability of class *c*, then the posterior probability of belonging to class *c *is given by the Bayes formula

$$p(c|x)=\frac{\pi_{c}p(x|c)}{p(x)}.$$

For classification purposes, we seek to assign samples to classes with the largest posterior probability. By maximizing the logarithm of the posterior distribution with the above assumption of *p*(*x|c*) distributed as *N*(*μ*_*c*_, Σ), we get

$$L_{c}=\log(p(x|c))+\log(\pi_{c})=x\Sigma^{-1}{\mu^{\prime }}_{c}-\frac{\mu_{c}\Sigma^{-1}{\mu^{\prime }}_{c}}{2}+\log(\pi_{c}),$$

which is a linear function in *x *directly corresponding to the LDA. In the case when covariance matrices are different for each class, i.e. ∑_*i *_≠ ∑_*j*_, we obtain a Quadratic Discriminant Analysis (QDA) which would be a quadratic function in *x*. Both LDA and QDA have been extensively used in practice with a fair share of success.

#### Support Vector Machines

The Support Vector Machines (SVM) is among the most recent significant developments in the field of discriminatory analysis [29]. In its very essence it is a linear classifier (just like logistic regression and LDA) as it directly seeks a separating hyperplane between classes which would have the largest possible margin. The margin is defined here as the distance between the hyperplane and the closest sample point. It is usually the case that there are several points called support vectors which are exactly one margin away from the hyperplane and on which the hyperplane is constructed. It is clear that as stated, SVM is of little practical use because most classification problems have no distinct separation between classes and, therefore, no such hyperplane exists. To overcome this problem, two extensions have been proposed in the literature: penalty-based and kernel methods.

The first approach relaxes the requirement of a "separating" hyperplane by allowing some sample points to be on the wrong side. It becomes a constrained optimization problem where the constraint is that the total distance of all misclassified points to the hyperplane is smaller than a chosen threshold *c*. The second approach is more elegant and frequently used. Since no linear separation between classes is possible in the original space, the main idea is to project into a higher dimensional space where such separation usually exists. It turns out that there is no need to specify such transformation *h*(*x*) explicitly and the knowledge of the kernel function is sufficient for optimization since kernel functions involve only the original non-transformed data which makes them easily computable

$$K(x_{i},x_{j})=h(x)'h(x).$$

The most popular choices for the kernel function are the *k *degree polynomial

$$K(x_{i},x_{j})={\left(1+{x^{\prime }}_{i}x_{j}\right)}^{k},$$

the Radial basis

$$K(x_{i},x_{j})=e^{\frac{-||x_{i}-x_{j}|{|}^{2}}{c}}$$

and the Neural Network kernel

$$K(x_{i},x_{j})=\tanh(k_{1}{x^{\prime }}_{i}x_{j}+k_{2}),$$

where *k, c, k*_1_, and *k*_2 _are parameters that need to be specified. SVMs enjoy the advantage in flexibility over most other linear classifiers. The boundaries are linear in a transformed high-dimensional space, but on the original scale they are usually non-linear which gives the SVM its flexibility whenever required.

#### Random Forests

Classification trees are particularly popular among medical researchers due to their interpretability. Given a new sample, it is very easy to classify it by going down the tree until one reaches the terminal node which carries the class assignment. Random Forests [3] take classification trees one step further by building not a single but multiple classification trees using different bootstrap samples (sampled with replacement). A new sample is classified by running it through each tree in the forest. One obtains as many classifications as there are trees. They are then aggregated through a majority voting scheme and a single classification is returned. The idea of bagging, or averaging multiple classification results, as applied in this context greatly improves the accuracy of unstable individual classification trees.

One of the interesting elements of Random Forests is the ability to compute unbiased estimates of misclassification rates on the fly without explicitly resorting to testing data after building the classifier. By using the samples which were left out of the bootstrap sample when building a new tree, also known as out-of-bag (OOB) data, RF runs the OOB data through the newly constructed tree and calculates the error estimate. These are later averaged out over all trees to obtain a single misclassification error estimate. This combination of bagging and bootstrap is sometimes called .632 cross-validation because roughly 2/3 of samples used for building each tree is really 1 - 1/*e *which is approximately .632. This form of cross-validation is arguably very efficient in the way it uses available data.

### Some commonly used dimension reduction techniques

For high-dimensional data, such as microarrays, where the number of samples is much smaller than the number of predictors (features), most of the classical statistical methodologies require a preprocessing step in which the dimensionality of data is reduced. The Principle Component Analysis (PCA) [30] and the Partial Least Squares (PLS) [31] are among two most popular methods for data dimension reduction. Of course, other more sophisticated dimension reduction techniques can be used as well. We use the PCA and PLS in a combination with logistic regression, LDA, QDA and Random Forests as illustrative examples.

Both PCA and PLS effectively reduce the number of dimensions while preserving the structure of the data. They differ in the way they construct their latent variables. The PCA selects the directions of its principal components along the axis of the largest variability in the data. It is based on the eigenvalue decomposition of an observed covariance matrix.

The PLS maximizes the covariance between dependent and independent variables trying to explain as much variability as possible in both dependent and independent variables. The very reason that it considers the dependent variable when constructing its latent components usually makes it a better dimension reduction technique than the PCA when it comes to classification problems.

## Authors' contributions

SD and SD designed the research and VP carried out simulations and wrote the first draft of the manuscript. All authors contributed to editing the final version.

## Availability

R code and additional examples are available through the supplementary website at http://www.somnathdatta.org/Supp/ensemble.
